# Dietary Glutamine Inclusion Regulates Immune and Antioxidant System, as Well as Programmed Cell Death in Fish to Protect against *Flavobacterium columnare* Infection

**DOI:** 10.3390/antiox11010044

**Published:** 2021-12-26

**Authors:** Congrui Jiao, Jiahong Zou, Zhenwei Chen, Feifei Zheng, Zhen Xu, Yu-Hung Lin, Qingchao Wang

**Affiliations:** 1 College of Fisheries, Huazhong Agricultural University, 1 Shizishan Street, Wuhan 430070, China; jcr-1998@webmail.hzau.edu.cn (C.J.); zjiahong@webmail.hzau.edu.cn (J.Z.); chenzhenwei@webmail.hzau.edu.cn (Z.C.); zhengfeifei@mail.hzau.edu.cn (F.Z.); zhenxu@mail.hzau.edu.cn (Z.X.); 2 Department of Aquaculture, National Pingtung University of Science and Technology, 1 Shuefu Road, Neipu, Pingtung 912, Taiwan

**Keywords:** antioxidant, apoptosis, autophagy, glutamine, immunity, mTOR signaling

## Abstract

The susceptibility of animals to pathogenic infection is significantly affected by nutritional status. The present study took yellow catfish (*Pelteobagrus fulvidraco*) as a model to test the hypothesis that the protective roles of glutamine during bacterial infection are largely related to its regulation on the immune and antioxidant system, apoptosis and autophagy. Dietary glutamine supplementation significantly improved fish growth performance and feed utilization. After a challenge with *Flavobacterium columnare*, glutamine supplementation promoted *il-8* and *il-1β* expression via NF-κB signaling in the head kidney and spleen, but inhibited the over-inflammation in the gut and gills. Additionally, dietary glutamine inclusion also enhanced the systematic antioxidant capacity. Histological analysis showed the protective role of glutamine in gill structures. Further study indicated that glutamine alleviated apoptosis during bacterial infection, along with the reduced protein levels of caspase-3 and the reduced expression of apoptosis-related genes. Moreover, glutamine also showed an inhibitory role in autophagy which was due to the increased activation of the mTOR signaling pathway. Thus, our study for the first time illustrated the regulatory roles of glutamine in the fish immune and antioxidant system, and reported its inhibitory effects on fish apoptosis and autophagy during bacterial infection.

## 1. Introduction

Unlike mammals, teleosts live in aquatic environments which are more conducive to bacterial growth [1,2], therefore potential pathogens could enter the bodies of fish across their mucosal epithelial barriers including the gills, gastrointestinal system or skin lesions [3,4,5]. Bacterial infection is one of the most common causes for animal diseases, both in mammals and other animals [6]. During infection, animals would firstly activate the innate immune system to defend against the invaders, and some animal species, mainly vertebrates, could further activate the adaptive immune system to efficiently clearing invading pathogens with a prolonged infecting period [7]. In particular, fish represent the earliest bony vertebrate to develop both innate and adaptive immune responses during evolution [8]. Reactive oxygen species (ROS) would also be induced by bacterial infection, however, the over-production of O^2−^ would cause oxidative damage to proteins, nucleic acids, and lipids [9]. Fish, like other animals, develop a cellular antioxidant defenses system to function in multiple situations including ROS scavenging, oxidative stress protection, and attenuation of membrane lipid peroxidation [10]. The major front-line antioxidant enzymes, such as superoxide dismutase (SOD, neutralizes superoxide radicals to H_2_O_2_), catalase (CAT) and glutathione peroxidase (GPx, neutralizes H_2_O_2_ to water), and small non-protein antioxidants (scavenges all active oxygen species directly) work in a cascade to protect cells from oxidative stress [11]. Moreover, the programmed cell death including autophagy and apoptosis are also related to an early evolution of defense against bacterial infection, for example, successful autophagic contributes to the clearance of the invading microbe and cell survival [12] while apoptosis helps to preserve the whole organism or the species from the spread of infection [13]. Autophagy is a fundamental eukaryotic process with multiple cytoplasmic homeostatic roles, recently expanded to include unique stand-alone immunological functions and interactions with nearly all parts of the immune system [14]. Autophagy has been identified to function as the effector or regulatory functions downstream of systems sensing danger signals/alarmins, also known as damage-associated molecular patterns (DAMP), such as ATP and self-DNA-containing complexes [15]. The contribution of autophagy is complicated by the fact that autophagy can be either protective or harmful, depending on the biological context [16]. Besides autophagy, apoptosis could also be induced upon endogenous receptor/ligand systems on the surface of the pathogen-infected cell via the activation of several pro-apoptotic proteins, e.g., caspases, and the inactivation of anti-apoptotic proteins, e.g., NF-κB or MAP-kinases [17]. In particular, the induction of apoptosis in epithelial or endothelial cells might break the epithelia/endothelial cell barrier and permit the bacteria to reach the sub-mucosa [18].

The immune responses, antioxidant capacity and programmed cell death of animals would be significantly affected by their nutritional status [19]. Normally, functional nutrients could perform a substrate role in the initial development of the immune cells and during an actual immune response so that the responding cells can divide and synthesize effector molecules [20]. Nutrients may perform direct regulatory actions on the leukocytes that respond to infectious challenges, and also perform indirect effects during bacterial infection via the modulation of the endocrine system [21,22]. Moreover, antioxidant nutrients could act as the safeguard against the accumulation of ROS and their elimination from the system to ensure redox homeostasis [23] via preventing lipid peroxidation, stopping the oxidative chain reaction in membranes and lipoproteins [24], and promoting the synthesis of antioxidant enzymes [25]. Additionally, nutrient starvation and specific nutrients have also been reported to regulate the apoptosis and autophagy in multiple kinds of cells [26,27]. Among all nutrients, amino acids are traditionally considered to compose proteins, and recently have been reported to function as direct signals to activate several signaling pathways and further play multiple functions [28]. Glutamine (Gln) is an abundant amino acid in blood which also functions as the most important fuel for intestinal tissue to support gut protein synthesis [29,30], regulate antioxidant [31] and immune system [32,33], promote cell proliferation [34], and delay apoptosis [35,36]. Glutamine has also been reported to activate several cell signaling-related kinases to play multiple functions [37]. For example, glutamine enhances intestinal cells proliferation via activating MAPKs [38], promotes protein synthesis by activating mTOR signaling [39,40], and improves cell survival by regulating heat shock proteins in the intestine [41,42]. In particular, glutamine functions in maintaining gut integrity in both humans and terrestrial animals by serving as a major energy substrate for the rapid development of enterocytes [43,44]. Approximately 70% of glutamine is degraded by rat and pig small intestines during the first pass [45,46] and glutamine deprivation induces autophagy and alters the mTOR and MAPK signaling pathways in porcine intestinal epithelial cells [47].

In fish, glutamine has also been reported to promote growth, enhance antioxidant capacity, promote intestinal function, improve healthcare function and even muscle flavor [48,49], however, there is little information about the regulatory mechanism. Unlike the normally twice duplicated genome during the evolution of vertebrates, a third genome duplication occurs in teleosts, i.e., fish-specific genome duplication (FSGD), which makes the teleost a research model to illustrate novel mechanism [50,51]. Recent studies have identified the FSGD in yellow catfish (*Pelteobagrus fulvidraco*) [52] and also the regulatory roles of apoptosis and autophagy in lipid metabolism of yellow catfish, for example, the apoptosis signaling pathways is reported to mediate Met-induced changes of hepatic lipid deposition and metabolism [53] and autophagy is involved in FA-induced TG accumulation and lipotoxicity in yellow catfish [54]. However, the apoptosis and autophagy responses during bacterial infection are rarely evaluated in teleosts, including yellow catfish. The columnaris disease, induced by *Flavobacterium columnare* (*F. columnare*) infection [55], is common in yellow catfish which exhibits skin lesions, fin erosion and gill necrosis with a high degree of mortality [56]. During *F. columnare* infection, yellow catfish could activate both innate immunity, such as the increased release of pro-inflammatory cytokines and reactive oxygen species (ROS) and adaptive immunity such as the secretion of immunoglobulins [56,57]. However, unlike most teleosts, no IgT/IgZ which is the executor of fish mucosal immunity exists in the genome of yellow catfish, and its mucosal immunity is far from knowledgeable [57,58]. Thus, the yellow catfish is a good experimental model to study the regulatory mechanism of immune and antioxidant systems, apoptosis and autophagy in teleost after bacterial infection. Accordingly, glutamine was supplemented to the diet of the yellow catfish for an 8-week rearing experiment in the present study, which was then infected with *F. columnare* to systematically evaluate the regulatory mechanism. This study made the first attempt to evaluate the influence of dietary glutamine inclusion on the apoptosis and autophagy responses in fish during bacterial infection.

## 2. Materials and Methods

### 2.1. Fish Husbandry

Yellow catfish of approximately 4.0 g, bought from a fish farm in Wuhan (Hubei, China), were maintained in independent circular fiberglass tanks (80 cm in diameter, 100 cm in column height) at the wet lab of Huazhong Agricultural University. The temperature of all water tanks was maintained at 24 ± 1 °C, and the peripheral speed of water was 8 L/min. Fish were fed twice per day with a commercial diet. All animal proceeding procedures were approved by the Institutional Animal Care and Use Committee of Huazhong Agricultural University.

### 2.2. Experimental Diet Preparation and Feeding

The composition of the basal diet is shown in Table 1. In the experimental diet, glutamine (1%) was added to the basal diet. At the beginning of the fish rearing experiment, yellow catfish were randomly assigned to 10 fish tanks, with 30 fish per tank. Fish in five tanks fed with the basal diet were set as the control group, while fish in another five tanks fed with the experimental diet were set as the experimental group. All other feeding and domestication conditions were the same in the two groups. After 60 days of the rearing experiment, fish within each tank were weighed and the diet used in each tank was calculated. Then fish growth performance and feed utilization were calculated, including final body weight (FBW), weight gain rate (WGR), specific growth rate (SGR) and feed conversion ratio (FCR). The parameters were calculated as the following equations which were determined according to previous reported methods [56]:

Weight gain rate (WGR, %) = 100 × [final body weight (g)-initial body weight (g)]/initial body weight (g);

Specific growth rate (SGR, %/day) = 100 × [ln (final body weight (g)) − ln (initial body weight (g))]/rearing period (days);

Feed conversion ratio (FCR) = dry feed intake (g)/[final body weight (g) − initial body weight (g)].

### 2.3. Bacterial Challenge Experiment

After the calculation of growth parameters, bacterial challenge test was conducted for fish in two groups. The water height of all 10 fish tanks was reduced to 20 cm, and then *F. columnare* medium was poured into tank at a final concentration of 5 × 10^5^ CFU/mL which was kept for 2 h to perform bacterial infection [56]. After bacterial challenge experiment, fresh water was added to all tanks and fish were reared under the same conditions. Fish in the first two tanks per group were sampled on days 0, 1 and 30 after exposure. During sampling, fish were first anesthetized via MS222 exposure (1:10,000), and blood was drawn from the tail vein. Serum was obtained after centrifugation at 3000× *g* for 20 min at 4 °C and stored at −80 °C until further analyses. Then the head kidney (HK), spleen, gill, gut, and liver were removed. Partial gills were stored in paraformaldehyde, and other tissue samples (gill, gut, spleen, liver, HK, and serum) were immediately frozen in liquid nitrogen and stored at −80 °C before analysis. Fish in the other three tanks per group were not sampled and calculated at the end of 30 days to determine mortality.

### 2.4. Histopathological Structure

The gill samples were shaped and then fixed with paraformaldehyde for 24 h. Each tissue was cut into an appropriate size and set into the embedding box. Then tissues were transferred to gradient ethanol for dehydration, xylene for transparent treatment, and then paraffin wax for embedding. The embedded tissue was trimmed and then installed on a microtome for sectioning. The 5 μm-thick tissue sections were used for hematoxylin-eosin (H.E.) staining following the manufacturer’s instructions. Stained glass slides were examined under an optical microscope (Olympus, DP72, Tokyo, Japan) equipped with a camera (Nikon E600, Nikon, Melville, NY, USA) and CellSens Standard Software (Olympus) to acquire images.

### 2.5. Enzyme Activity Assay

Total antioxidant capacity (T-AOC) and the activities of superoxide dismutase (SOD), catalase (CAT) and glutathione peroxidase (GPx) in serum were analyzed with commercially available assay kits (Nanjing Jiancheng Bioengineering Institute, Nanjing, China). T-AOC was assayed as the equal amount (mM) of trolox solution that could stop the oxidization of 2,2′-azino-bis(3-ethylbenzthiazoline-6-sulfonic acid (ABTS) to green ABTS^+^ by reactive oxygen species (ROS). SOD activity (units per milliliter serum) was measured spectrophotochemically with each unit defined as the amount of enzyme necessary to inhibit the reduction of water-soluble tetrazolium salt by 50%. CAT activity (units per milliliter serum) was measured with one unit defined as the amount of activity required to transform 1 μmol of H_2_O_2_ per min. GPx activity was measured with one unit defined as the amount of GPx required to consumed 1 μmol of glutathione per min.

### 2.6. RNA Extraction and cDNA Preparation

Total RNA was extracted from gill, gut, head kidney, and spleen samples using Trizol (Invitrogen, Carlsbad, CA, USA) as described by the manufacturer. Briefly, samples were mechanically disrupted in 1 mL of Trizol using a homogenizer. Then, 200 mL of chloroform was added, and the suspension was centrifuged at 12,500 r/pm for 15 min. The clear upper phase was recovered, and mixed with an equal volume of isopropanol. The mixture was allowed to stand sufficiently to precipitate, and then centrifuged. The pellet was washed with ethanol, and added with appropriate DEPC-treated water to dissolve the precipitate. The spectrophotometer was used to determine the concentration and purity of RNA sample, controlling the its absorbance range with OD260/280 values between 1.8–2.2. RNA integrity was then checked by 1.0% agarose gel electrophoresis. DNase treatment was performed with 5× gDNA Digester Mix (Yeasen, China) according to the manufacturer’s instructions and cDNA was synthesized from 1 μg of total RNA using 4× Hifair^®^ III SuperMix plus (Yeasen, China). The synthesized cDNA was stored at −20 °C until use.

### 2.7. Quantitative Real-Time Polymerase Chain Reaction (PCR) Analysis

To assess the transcription levels of multiple genes, real-time quantitative PCR was performed on a 7500 Real-time PCR System (Applied Biosystems) using EvaGreen 2× qPCR Master Mix (Abm) with a 10 μL reaction volume containing 5 μL of 2× qPCR MasterMix, 1 μL of template (200 ng/μL), 0.15 μL of each forward and reverse primer (10 μM), and 3.7 μL of H_2_O. The cycling conditions for all samples were: 95 °C for 30 s, then 40 cycles at 95 °C for 1 s and 58 °C for 10 s. Melt curve analysis was performed after the amplification cycle to confirm the accuracy of each amplicon. The expression of the target gene was calculated using the 2^−ΔΔCt^ method after normalized relative to the relative expression of ef-1α, which has been tested to be stable under the current experimental conditions. Table 2 lists the primer information used in this study.

### 2.8. TdT-Mediated dUTP Nick-End Labeling (TUNEL) Assays

TdT-mediated dUTP nick-end labeling (TUNEL) assay was conducted to precisely detect apoptotic cells with the one-step TUNEL Apoptosis Assay kit (C1088, Beyotime, Nanjing, China) following the manufacturer’s instructions. Briefly, gills were dewaxed in xylene for 5–10 min, and then changed to fresh xylene for another 5–10 min. Then, tissues were immersed into anhydrous ethanol for 5 min, 90% ethanol for 2 min, 70% ethanol for 2 min, and distilled water for 2 min. Tissues were treated with DNase-free proteinase K (Beyotime ST535, 20 μg/mL) at 20–37 °C for 15–30 min and then washed with PBS 3 times to remove excess proteinase K. Afterwards, the labelling reaction was performed using a labelling solution containing terminal deoxynucleotidyl transferase, buffer, and fluorescein dUTP at 37 °C for 60 min in a humidity chamber. Following incubation, excess labelling solution was washed off smears with PBS, and then cell smears were mounted with fluorescent microscopy mounting solution. Images were captured and analyzed using a CCD camera (Olympus CKX41, Tokyo, Japan).

### 2.9. Western Blotting Analysis

Tissues was homogenated with RIPA buffer which has been added with proteinase inhibitor and phosphorylated-protease inhibitor to extract protein. Protein concentration in all groups were calculated with an enhanced BCA Protein Assay Kit (P0010, Beyotime Tech, Shanghai, China) and then adjusted to same level. The same amounts of protein in each group were separated on SDS-PAGE, and then transferred to PVDF membranes. The membranes were blocked in 6% skim milk at room temperature for 2 h and then incubated at 4 °C with the relevant primary antibodies overnight. Then, membranes were incubated with the appropriate HRP-labeled secondary antibodies for 1 h at room temperature. Immunoreactive bands were visualized using ECL reagent under GE Amersham Imager 600 Imaging System (GE Healthcare, Boston, MA, USA) and protein levels were digitized using ImageQuant TL software (GE Healthcare). Primary antibodies for phospho-S6 (Ser 235) and LC3B-II were purchased from Cell Signaling Technology (Beverly, MA, USA). The primary antibody for caspase-3 was purchased from Abcam (Cambridge, UK). Primary antibody for β-actin was purchased from ABClonal Biotechnology (Wuhan, China).

### 2.10. Statistical Analysis

All statistical analyses were performed using SPSS 17.0. Data about fish growth performance and feed utilization was analyzed using student’s *t*-test [56,59]. All gene-expression and histological calculation results were tested for normality using the Shapiro–Wilk-W’s test, and normally distributed data were analyzed by factorial (two-way) analysis of variance (ANOVA) to determine the main effects of sampling days (D) and feeding treatment (T), and their interactions (D*T). When significant D*T were observed, data were analyzed by one-way ANOVA followed by Tukey’s multiple range tests to inspect differences among all the data. When the significance is only with the main effects of D or T, the data were analyzed by the two-way ANOVA followed by Tukey’s multiple range tests to assess the main effects of D or T only. Differences were considered significant when *p* < 0.05. All data were expressed as mean ± standard deviation of the mean (SD), except the specific statement.

## 3. Results

### 3.1. Dietary Glutamine Significantly Increased Growth Performance, Feed Utilization and the Disease Resistance of Yellow Catfish

In the present study, dietary glutamine inclusion showed a significant promoting effect on the growth performance of yellow catfish, with higher final body weight (Table 3). The weight gain rate and specific growth rate of yellow catfish in the glutamine group (530.44 ± 2.29% & 1.43 ± 0.01%/day, respectively) were significantly higher than those in the control group (507.74 ± 6.84% & 1.40 ± 0.01%/day, respectively), while the feed conversion rate in the glutamine group (0.94 ± 0.04) was significantly lower than that in the control group (1.07 ± 0.01).

In order to detect the disease resistance ability of yellow catfish after dietary glutamine supplementation, fish in two groups were challenged with *F. columnare*. After the immersed infection of *F. columnare*, yellow catfish fed with glutamine supplementation showed a significantly higher survival rate (92%) than fish fed with control diet (74%) (Table 3).

### 3.2. Glutamine Differentially Regulated Cytokines Expression in Multiple Fish Tissues after Bacterial Infection

**Figure 1:**
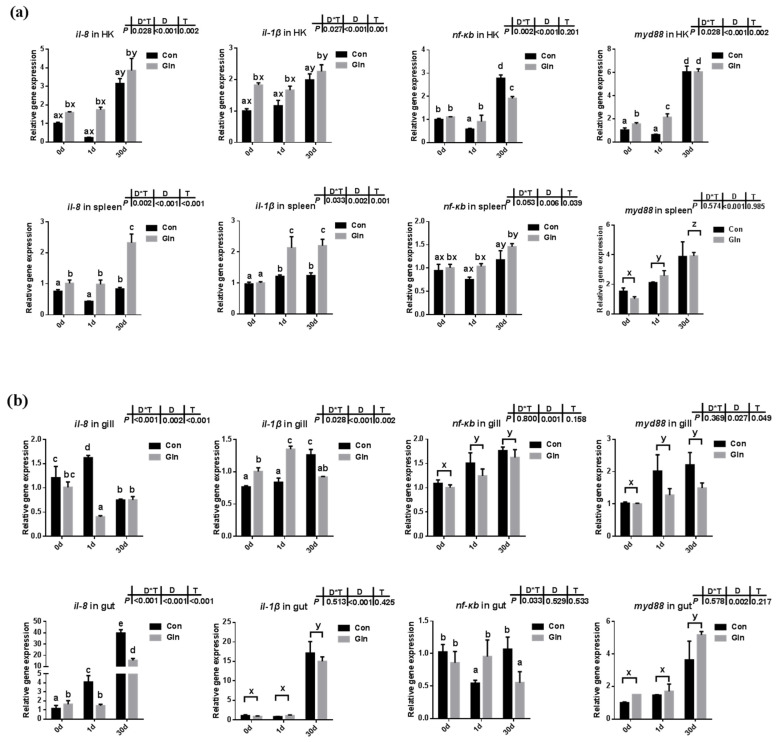
The effects of dietary glutamine on the inflammatory responses in systematic and mucosal immune tissues of yellow catfish during *F. columnare* infection. The relative mRNA expression levels of pro-inflammatory cytokines (*il-8*, *il-1β*) and genes involved in NF-κB signaling pathway (*nf-κb*, *myd88*) in the head kidney, spleen (**a**), and gill, gut (**b**) of yellow catfish fed with control diet (Con) and glutamine supplementation diet (Gln) at 0 d, 1 d and 30 d after *F. columnare* infection. All data was analyzed with two-way ANOVA and data are means ± SD (*n* = 3). When significant D*T were observed (*p* < 0.05), data were analyzed by one-way ANOVA followed by Tukey’s multiple range tests to inspect differences among all the data, with “a–e” to indicate the significant difference. When the significance is only with the main effects of D or T, “a–e” and “x–z” were labeled to indicate the significant difference among groups in D or T, respectively.

The expression levels of cytokines along with genes involved in NF-κB/MyD88 signaling pathway were evaluated in both systematic (Figure 1a) and mucosal-associated immune tissues (Figure 1b). In the systematic immune organ, the expression of *il-8* and *il-1β* in the head kidney and spleen were significantly increased at 30 d after infection. Accordingly, the expression of *nf-κb* and *myd88* was also increased in the head kidney at 30 d. Moreover, compared to control group, dietary glutamine promoted the expression of *il-8* and *il-1β* in the head kidney, and also increased the expression of *nf-κb* and *myd88* at 1 d. In the spleen, interactions between sampling days (D) and dietary treatment (T) were found on the expression of *il-8* and *il-1β*. In the control group, *il-8* expression was increased at 30 d, and *il-1β* expression was increased at both 1d and 30 d. Dietary glutamine supplementation further promoted the expression of *il-8* and *il-1β* at 1 d and 30 d in the spleen. The expression of *nf-κb* and *myd88* significantly increased with prolonged infection period, while dietary glutamine further promoted the expression of *nf-κb* in the spleen.

In the mucosal-associated immune tissues, there was also a significant interactive effect of sampling days and dietary treatment were found on the expression of *il-8* (*p* < 0.001) and *il-1β* (*p* = 0.002) in the gill, and the expression of *il-8* in the gut (*p* < 0.001). In the control group, the expression of *il-8* was upregulated at 1 d in the gill and gut, but only upregulated at 30 d in the gut. Dietary glutamine significantly decreased the expression of *il-8* in the gill at 1 d and its expression in the gut at 1d and 30 d. In the control group, the expression of *il-1β* in the gill and gut significantly increased at 30 d, and the inhibitory effects of dietary glutamine was detected at 30 d. The expression of *nf-κb* in the gill and gut was significantly upregulated at 1d and 30 d compared to that at 0 d. Similarly, the expression of *myd88* in the gut was significantly upregulated at 30 d compared to that at 0 d, and dietary glutamine exhibited inhibitory effects on *myd88* expression in the gill at 30 d.

### 3.3. Glutamine Enhanced Fish Antioxidant Capacity against F. columnare Infection

**Figure 2:**
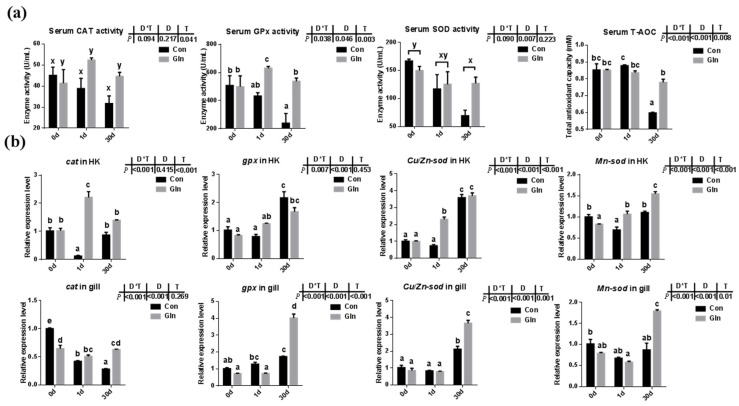
The effects of dietary glutamine on the antioxidant system of yellow catfish during *F. columnare* infection. (**a**) The enzyme activities of catalase (CAT), glutathione peroxidase (GPx), superoxide dismutase (SOD) along with total antioxidant capacity in serum of yellow catfish in both control group (Con) and glutamine supplementation group (Gln) at 0 d, 1 d and 30 d after *F. columnare* infection (*n* = 6). (**b**) The relative mRNA expression level of *cat*, *gpx*, *Cu/Zn-sod* and *Mn-sod* in head kidney and gill of yellow catfish in all treatments (*n* = 3). All data was analyzed with two-way analysis of variance (ANOVA) and data are means ± SD. When significant D*T were observed (*p* < 0.05), data were analyzed by one-way ANOVA followed by Tukey’s multiple range tests to inspect differences among all the data, with “a–e” to indicate the significant difference. When the significance is only with the main effects of D or T, “a–e” and “x–z” were labeled to indicate the significant difference among groups in D or T, respectively.

Fish antioxidant capacity during *F. columnare* infection was also significantly affected by dietary glutamine inclusion. As shown in Figure 2a, dietary glutamine inclusion significantly enhanced serum CAT activity. Moreover, serum GPx activity at 1d and 30 d was also increased after dietary glutamine inclusion. Serum SOD activity was only affected by sampling days but not by dietary treatment. In all, the serum total antioxidant capacity (T-AOC) showed a significant increase at 30 d after glutamine inclusion.

### 3.4. Glutamine Protected Fish Gill Structures against F. columnare Infection

**Figure 3:**
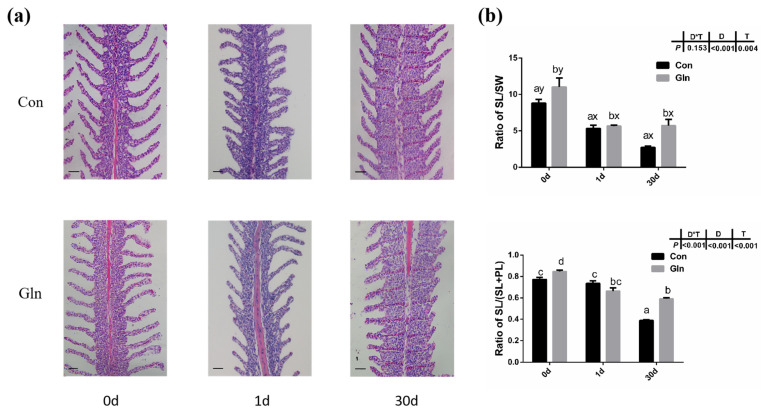
The effects of dietary glutamine on gill histological structures of yellow catfish during *F. columnare* infection. (**a**) The H.E. staining results of yellow catfish gill before and after *F. columnare* infection. Scale bars, 100 μm. (**b**) The ratio of secondary lamellae length (SL) to secondary lamellae width (SW) and the ratio of secondary lamellae length (SL) to the sum of primary lamellae length (PL) and SL. All data were analyzed with two-way ANOVA and data are means ± SD (*n* = 12). When significant D*T were observed (*p* < 0.05), data were analyzed by one-way ANOVA followed by Tukey’s multiple range tests to inspect differences among all the data, with “a–e” to indicate the significant difference. When the significance is only with the main effects of D or T, “a–e” and “x–z” were labeled to indicate the significant difference among groups in D or T, respectively.

The gill plays an important role in the respiration, ion exchange and immune responses of fish, and the gill is the main infecting target tissue of *F. columnare*. In the present study, *F. columnare* infection significantly affected the histological structures of gill and the space between adjacent gill lamellae was occluded at 1 d (Figure 3). Moreover, the ratio of secondary lamellae length (SL) to secondary lamellae width (SW) and the ratio of SL to (SL and primary lamina length (PL)) were also significantly decreased after bacterial infection at both 1 d and 30 d. Dietary glutamine supplementation significantly alleviated the gill’s structure induced by *F. columnare* infection, with an integrated structure of gill lamellar in glutamine supplementation group fish at 1d and 30 d. Moreover, the ratio of SL to SW and the ratio of SL to (SL+PL) were also significantly higher in the gill of yellow catfish with dietary glutamine supplementation than fish fed with the control diet at 30 d.

### 3.5. Glutamine Inhibited the Apoptosis of Fish Gill during Bacterial Infection

**Figure 4:**
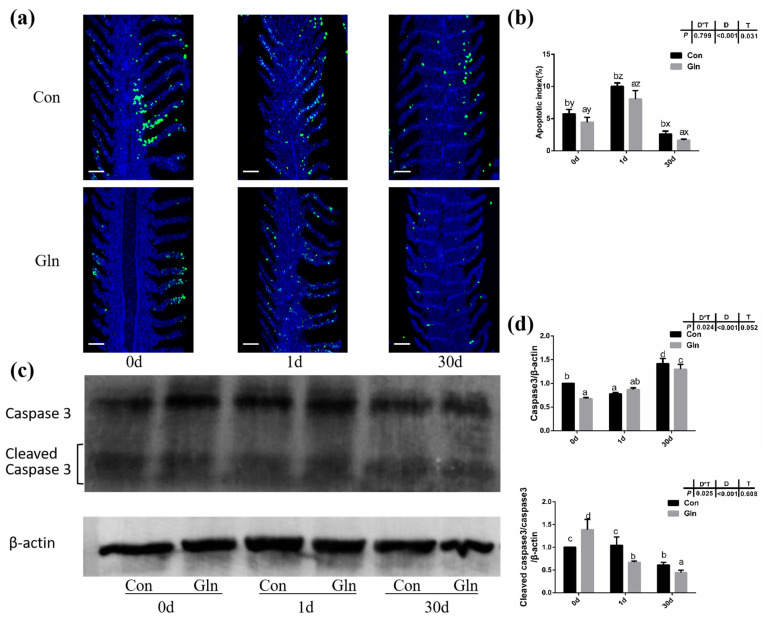
The effects of dietary glutamine on the gill apoptosis of yellow catfish during *F. columnare* infection. (**a**) TUNEL assay of apoptotic cells in the gill of yellow catfish in both the control group (Con) and glutamine supplementation group (Gln) at 0 d, 1 d and 30 d after *F. columnare* infection. Stained for apoptosis cells (green), nuclei are stained with DAPI (blue). Scale bar, 100 μm. (**b**) The apoptotic index of gill cells in yellow catfish among all treatments (*n* = 12). (**c**) The Western blotting analysis of caspase-3 and cleaved caspase-3 in yellow catfish among all six treatments. (**d**) The ratio of caspase-3 protein level/β-actin protein level and the ratio of cleaved caspase-3 protein level/caspase-3 protein level/β-actin protein level (*n* = 6). When significant D*T were observed (*p* < 0.05), data were analyzed by one-way ANOVA followed by Tukey’s multiple range tests to inspect differences among all the data, with “a–e” to indicate the significant difference. When the significance is only with the main effects of D or T, “a–e” and “x–z” were labeled to indicate the significant difference among groups in D or T, respectively.

**Figure 5:**
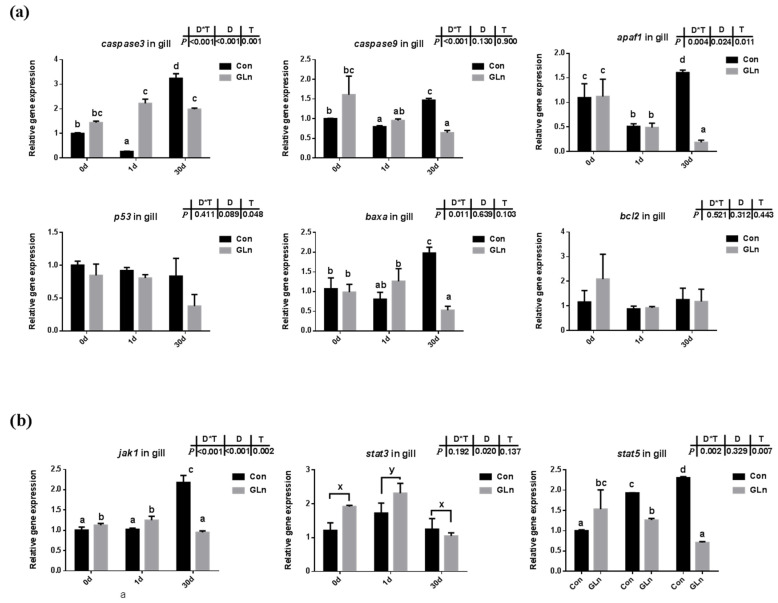
The effects of dietary glutamine on the apoptosis of yellow catfish during *F. columnare* infection along with the JAK-STAT signaling pathway. (**a**) The relative mRNA expression level of *caspase-3*, *caspase-9*, *apaf1*, *p53*, *baxa*, *bcl2* in gill of yellow catfish in both control group (Con) and glutamine supplementation group (Gln) at 0 d, 1 d and 30 d after *F. columnare* infection. (**b**) The relative mRNA expression level of *jak1*, *stat3*, *stat5* in gill of yellow catfish in all treatments. All data were analyzed with two-way ANOVA and data are means ± SD (*n* = 3). When significant D*T were observed (*p* < 0.05), data were analyzed by one-way ANOVA followed by Tukey’s multiple range tests to inspect differences among all the data, with “a–e” to indicate the significant difference. When the significance is only with the main effects of D or T, “a–e” and “x–z” were labeled to indicate the significant difference among groups in D or T, respectively.

TUNEL analysis was adopted to determine the effects on the apoptosis in fish gills and results indicated that the apoptosis within fish gill significantly increased at 1d but then decreased at 30 d after bacterial infection (Figure 4a,b). Dietary glutamine supplementation showed a significant inhibitory role on the apoptosis of the gill. Considering that apoptosis was executed by caspase-family member proteins and regulated by different regulators, both the protein level and gene expression level of caspase-3 were evaluated, along with the expression of other related genes. As shown in Figure 4c,d, the protein levels of caspase-3 significantly increased at 30 d after bacterial infection, while dietary glutamine supplementation significantly decreased the caspase-3 protein level at both 1 d and 30 d. However, the ratio of cleaved caspase-3/caspase-3 was not increased but even decreased at 30 d after infection. Dietary glutamine increased the ratio of cleaved caspase-3/caspase-3 at 0 d but decreased this ratio after infection both at 1 d and 30 d. Additionally, the expression levels of *caspase-3*, *caspase-9*, *apaf1* and *baxa* were upregulated at 30 d in the control group (Figure 5a). Dietary glutamine supplementation showed significant inhibitory roles on the expression of *caspase-3*, *caspase-9*, *apaf1* and *baxa* at 30 d, while it increased the expression of *caspase-3* at 1 d.

The apoptosis has been reported to be under regulation by JAK-STAT signaling pathway, and the expression levels of related genes were evaluated in the present study (Figure 5b). Results indicated that interactions between sampling days and dietary treatment were found on the expression of *jak1* (*p* = 0.002) and *stat5* (*p* = 0.007). In the control group, the expression levels of these genes were significantly higher at 30 d than at 0 d. Dietary glutamine supplementation significantly decreased the expression of *jak1* at 30 d, and *stat5* at 1 d and 30 d. However, the expression of *stat3* was only affected by different sampling days and it was significantly upregulated at 1 d.

### 3.6. Glutamine Inhibited the Autophagy of Yellow Catfish via the Activation of mTOR Signaling

**Figure 6:**
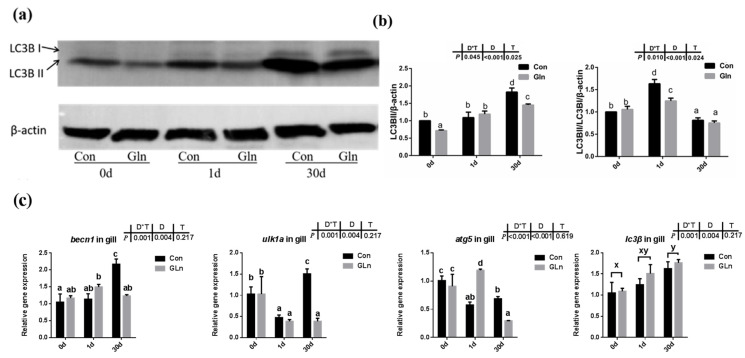
The effects of dietary glutamine on the autophagy of yellow catfish during *F. columnare* infection. (**a**) Western blotting analysis of LC3B I and LC3B II in yellow catfish in both control group (Con) and glutamine supplementation group (Gln) at 0 d, 1 d and 30 d after *F. columnare* infection. (**b**) The ratio of LC3B II protein level/β-actin protein level and the ratio of LC3B II/LC3B I/β-actin protein level in yellow catfish among all treatments (*n* = 6). (**c**) The mRNA expression of *becn1*, *ulk1a*, *atg5* and *lc3β* in the gill of yellow catfish among all treatments. All data were analyzed with two-way ANOVA and data are means ± SD (*n* = 3). When significant D*T were observed (*p* < 0.05), data were analyzed by one-way ANOVA followed by Tukey’s multiple range tests to inspect differences among all the data, with “a–e” to indicate the significant difference. When the significance is only with the main effects of D or T, “a–e” and “x–z” were labeled to indicate the significant difference among groups in D or T, respectively.

In order to determine the effects on autophagy, both the protein level of LC3B-II protein and the mRNA expression of related genes were evaluated. Results indicated that there was a significant interactive effect of sampling days and dietary treatment on the protein levels of LC3B-II (*p* = 0.002) and the ratio of LC3B-II/LC3B-I (*p* = 0.002) (Figure 6a,b). The protein level of LC3B-II significantly increased with prolonged infection period and highest level was detected at 30 d, however, the ratio of LC3B-II/LC3B-I showed the highest value at 1 d. Moreover, dietary glutamine significantly inhibited the protein levels of LC3B-II at 0 d and 30 d, and also significantly decreased the ratio of LC3B-II/LC3B-I at 1 d. Moreover, the mRNA expression levels of *becn1*, *ulk1a* and *atg5* were interactively affected by sampling days and feeding treatment. Dietary glutamine significantly decreased the expression of *becn1*, *ulk1a* and *atg5* at 30 d. The expression of *lc3β* was only affected by bacterial infection days but not feeding treatment, and its expression level was upregulated after bacterial infection.

**Figure 7:**
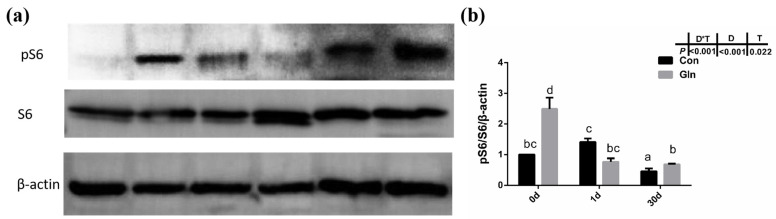
The effects of dietary glutamine on the mTOR signaling pathway activation of yellow catfish during *F. columnare* infection. (**a**) Western blotting analysis of pS6 and S6 in yellow catfish in both control group (Con) and glutamine supplementation group (Gln) at 0 d, 1 d and 30 d after *F. columnare* infection. (**b**) The ratio of pS6/S6/β-actin protein level in yellow catfish among all treatments. All data was analyzed with two-way ANOVA and data are means ± SD (*n* = 6). Mean values with different letters indicated significant differences among groups, *p* < 0.05.

Given that autophagy could be regulated by mTOR signaling pathway in mammals, the activation status of the mTOR signaling pathway in yellow catfish were evaluated by the phosphorylation of S6. Results showed that the phosphorylation level of S6 (p-S6) was significantly increased by dietary glutamine both at 0 d and 30 d (Figure 7), indicating the activation of mTOR signaling pathway. Thus glutamine might activate the mTOR signaling pathway to inhibit the autophagy responses during bacterial infection.

## 4. Discussion

In the present study, after a 60-day feeding experiment, dietary glutamine supplementation significantly improved the growth performance of yellow catfish with higher final body weight (29.38 ± 0.09 g) than the control fish (28.04 ± 0.30 g) (Table 3). The increased body weight should be mainly attributed to higher protein synthesis [60], and protein synthesis in fish is mainly regulated by the mTOR signaling pathway [59]. In the present study, glutamine supplementation also significantly elevated the activation of the mTOR signaling pathway at the end of the feeding experiment (0 d), indicated by the increased phosphorylation of S6 (Figure 7). Thus glutamine activated TOR to promote fish protein synthesis, which resulted in the increased fish body weight. Additionally, dietary glutamine supplementation also improved feed utilization, which may be related to the enhanced nutrient-absorptive role, as glutamine has been reported to be one of the most important energy sources for enterocytes [46]. In order to study the protective effect of glutamine against bacterial infection, yellow catfish were infected with *F. columnare* via soaking, which is the pathogen responsible for columnaris disease [61]. The structure of the gill tissue, which is the target organ of *F. columnare*, in yellow catfish was significantly affected after *F. columnare* infection in the present study, showing occluded space between adjacent gill lamellae and decreased length of the secondary lamellae, which was similar to our previous study [56]. Glutamine has been reported to promote the histological structures of multiple mucosal tissues in both mammals and teleosts. In mammals, glutamine can positively affect gut health by supporting the gut microbiome and gut mucosal wall integrity [62], reduce mortality in rodents with sepsis [63], and attenuate lipopolysaccharide-induced acute lung injury via enhanced GSH synthesis [64,65]. In teleosts, earlier studies have also reported that dietary glutamine has a positive effect on improving the integrity of mucosal tissues such as gut in channel catfish [66] and hybrid striped bass (*Morone chrysops, Morone saxatilis*) [67]. Dietary nutrients such as valine [68], tryptophan [69] and isoleucine [70] affected the gill health status of young grass carp (*Ctenopharyngodon idella*) during normal feeding status while other nutrients such as vitamin E [71], phosphorus [72], pyridoxine [73], and choline [74] have also been reported to correlate with the incidence of rotten gill and gill morphology of grass carp during *F. columnare* infection. In the present study, dietary glutamine supplementation significantly alleviated the damage during bacterial infection, as the ratio of SL to SW and the ratio of SL to (SL+PL) were significantly increased with dietary glutamine supplementation at 30 d. All these results indicate that the addition of dietary glutamine helps yellow catfish to maintain the integrity of mucosal tissues and resist *F. columnare* infection, resulting in decreased mortality. Additionally, the protective roles of glutamine on fish gill structures significantly promote the growth performance of yellow catfish.

Teleosts were the first bony vertebrate to develop both innate and adaptive immunity, whose immune system contains both systemic immune organs (e.g., head kidney, spleen) and mucosal-associated immune tissues (e.g., skin, gill, gut) [75]. In the present study, the expression of *il-8* and *il-1β* were significantly upregulated in the head kidney and spleen at 30 d after infection, which was accompanied with the increased expression of *nf-κb* and *myd88*. Similar results were found in the gill and gut with the increased expressions of *il-8*, *il-1β*, *nf-κb* and *myd88* mainly at 30 d, indicating that *F. columnare* activates the NF-κB/Myd88 signaling pathway to stimulate the release of cytokines in both systematic and mucosal immune tissues, which was also consistent with our previous results [56]. The appropriate release of pro-inflammatory cytokines in immune organs is important for bacterial clearance and animal health [76]. Dietary glutamine supplementation promoted quicker inflammatory responses in the head-kidney and spleen at 1 d to fight against bacterial infection, indicated by the increased expression of *il-8* and *il-1β*. However, excess inflammation is also damaged to animal health, especially in the mucosal tissues that contained not only leukocytes but also more epithelial cells [77]. The limited inflammatory response and appropriate recovery of epithelial cells within those mucosal tissues are important to fish health. Here, dietary glutamine supplementation significantly decreased the expression of *il-8* in the gill at 1 d, and the expression of *il-1β* at 30 d, which contributed to the decreased mortality after bacterial infection. In mammals, treatment with glutamine before and after ischemia significantly attenuated the increases of TNF-α and CINC-1 levels in perfusate during ischemia-reperfusion induced acute lung injury (*p* < 0.05) [78]. Similarly, in the gut, the expression of *il-8* was significantly decreased by dietary glutamine supplementation at 1 d and 30 d, which indicated that the inflammation was significantly limited in a prolonged infection period. The inhibitory roles of glutamine on excess inflammation of gut are in agreement with previous reports in turbot which showed alleviation on the enteropathy induced by dietary soybean meal [79]. Glutamine has also been reported to be effective in protecting against H_2_O_2_-induced oxidative stress in carp intestinal epithelial cells [80]. In fact, the cellular antioxidant defense systems including antioxidant enzymes and small non-protein antioxidants in fish are also involved in the defense against bacterial infection. In the present study, the expression of representative antioxidant enzymes in the head kidney and gill were significantly decreased after dietary glutamine inclusion, indicating the lower oxidative stress. However, during *F. columnare* infection, the mRNA expression of *sod* (including *Mn-sod*, *Cu/Zn-sod*), *cat*, and *gpx* in the head kidney and gill were also significantly increased at 1 d and 30 d with dietary glutamine inclusion. Moreover, the serum enzyme activities of CAT and GPx were also significantly enhanced after dietary glutamine inclusion, resulting in the higher T-AOC in serum. The protective roles of glutamine in enhancing antioxidant capacity against various stresses has also been reported in other species such as mirror carp (*Cyprinus carpio* L.) [81], half-smooth tongue sole (*Cynoglossus semilaevis* Günther) [82], and sea cucumber (*Apostichopus japonicus* Selenka) [83].

As mentioned above, animals activate the immune and antioxidant system to execute the clearance of infecting microorganisms. However, if these immune responses fail to fight infection, the programmed cell death pathway can be activated to remove the infected cells from the organism [84]. Type I programmed cell death or apoptosis is critically important for the survival of multicellular organisms by getting rid of damaged or infected cells that may interfere with normal functions [85,86]. Considering the gill remains the main target organ of *F. columnare* infection [87] and the affected histological gill structures after infection, the apoptosis within fish gill was evaluated by a TUNEL assay in the present study. Bacterial infection significantly increased cell apoptosis in the gills of yellow catfish indicated by the TUNEL assay. Accompanied with this, both the protein level of caspase-3 and the mRNA expression of related genes including *caspase-3*, *caspase-9*, *apaf-1*, *p53* and *baxa* also significantly increased after infection. This was in accordance with previous studies in grass carp, which also detected the increased apoptosis in gill during *F. columnare* infection [73] and in the red blood cells during *Aeromonas hydrophila* infection [88]. In this study, dietary glutamine supplementation significantly reduced the ratio of apoptotic cells in the gill after bacterial infection, indicated by the TUNEL assay. In mammals, glutamine has also been found to have anti-apoptotic effects in rat intestinal epithelial (RIE-1) cell [89] and respiratory tissue (lung) [90]. In one former study, the metabolites of glutamine were reported to prevent hydroxyl radical-induced apoptosis through inhibiting mitochondria and calcium ion involved pathways in fish erythrocytes [91]. Thus it is reasonable that glutamine showed a protective role against apoptosis in fish gill in the present study. In order to further illustrate the regulatory mechanism of glutamine on apoptosis, the genes expression involved in the JAK-STAT signaling pathway were evaluated, which has been identified as one of the principal pathway responsible for apoptosis in mammals [92]. JAKs including JAK1, JAK2, JAK3, and TYK2, which function as the membrane receptors, can be activated upon preferential ligand binding and then recruits and phosphorylates STATs, preferentially STAT3 and STAT5, on their tyrosine residues [93]. Genes involved in the JAK-STAT signaling pathway have been identified in fish and also significantly affected by nutrients such as choline [74]. Our previous study also identified the regulatory role of the JAK-STAT signaling pathway on fish apoptosis during bacterial infection, which could be affected by herbal extracts [94]. In the present study, the expression of related genes including *jak1* and *stat5* were significantly increased at 30 d after *F. columnare* infection, while dietary glutamine supplementation significantly decreased their expression at 30 d. Thus glutamine might inhibit the JAK-STAT signaling pathway to decrease the apoptosis in fish gills after bacterial infection.

Besides apoptosis, autophagy, also called Type II programmed cell death, is a form of self-defense of cells against pathogenic micro-organisms, and has also been found to function during bacterial infection [95]. Autophagy has been reported to correlate with animal immune responses, as it can be used as a general factor to affect the basic functions of cells, and can also function as a special effector to regulate immune function [12]. Autophagy also plays an important role in teleost fish, and it has been reported to be involved in the lipid metabolism in gut epithelial cells of yellow catfish [96]. LC3B supported the extension and closure of phagocytes to form a double-membrane autophagosome, thereby enabling the autophagy mechanism towards maturity [97]. In the present study, both the protein level of LC3B-II and the ratio of LC3B-II/LC3B-I protein in yellow catfish was significantly increased after *F. columnare* infection. However, glutamine significantly inhibited the protein level of LC3B-II and the ratio of LC3B-II/LC3B-I protein, indicating the inhibitory effects on autophagy. Besides LC3B, other factors such as becn1, ulk1a and atg5 have also been reported to participate in the fish autophagy process [98]. In the present study, the expression of *ulk1a* and *atg5* were also significantly downregulated after glutamine supplementation at 30 d, confirming the inhibitory role of glutamine on the autophagy of yellow catfish. In order to illustrate the regulatory mechanism of glutamine on autophagy, the activation level of TOR signaling pathway was determined by the phosphorylation level of S6 (p-S6), which has been reported to function as a regulator in fish autophagy [99]. Dietary inclusion of glutamine significantly increased the phosphorylation level of S6 (p-S6), indicating the higher activation level of mTOR signaling pathway. Thus glutamine may activate the TOR signaling pathway, which functions in the inhibition of autophagy in yellow catfish after *F. columnare* infection. The protective roles of glutamine in the present study are similar to earlier reports on methionine-chelated Zn, which promotes anabolism by integrating the mTOR signal and autophagy pathway [100]. Taken together, this provides direct evidence that bacterial infection causes autophagy in the fish itself, and that glutamine suppresses autophagy via activating the TOR signaling pathway.

## 5. Conclusions

In conclusion, our results emphasized the effect of glutamine on the growth promotion and disease resistance of yellow catfish, and initially explored the functions of glutamine on fish somatic cell apoptosis and autophagy during bacterial infection. The discovery highlights the importance of functional nutrients in fish immune response, and may lay the foundation for the research of fish nutrition metabolism and anti-bacterial disease treatment.

## Figures and Tables

**Table 1:** Formulation and proximate composition of the basal diet (% dry matter).

Ingredient	%	Ingredient	%
Fish meal	5	Vitamin premix ^a^	1
Wheat gluten meal	8	Mineral premix ^b^	1.5
Corn gluten meal	14	Monocalcium phosphate	1.5
Soybean meal	29	Choline chloride	0.5
Fish oil	2.5	Ethoxy quinoline	0.05
Soybean oil	2.5	Sodium alginate	2
Soy lecithin	1		
Wheat meal	31.45		
Proximate composition (%)			
Crude protein	39.62		
Crude lipid	7.49		

^a^ Vitamin premix (mg/kg dry diet): vitamin A 10, vitamin D 0.05, vitamin E 400, vitamin K 40, vitamin B_1_ 50, vitamin B_2_ 200, niacin 500, vitamin B_6_ 50, biotin 5, folic acid 15, vitamin B_12_ 0.1, vitamin C 1000, inositol 2000, choline 5000. ^b^ Mineral premix (mg/kg dry diet): FeSO_4_·7H_2_O 372, CuSO_4_·5H_2_O 25, ZnSO_4_·7H_2_O 120, MnSO_4_·H_2_O 5, MgSO_4_ 2475, NaCl 1875, KH_2_PO_4_ 1000, Ca(H_2_PO_4_)_2_ 2500.

**Table 2:** Primer used in the present study.

Gene Name AN	Accession No.	Forward Sequence	Reverse Sequence
Inflammatory cytokines			
*il-8*	KY218792.1	CACCACGATGAAGGCTGCAACTC	TGTCCTTGGTTTCCTTCTGG
*il-1* *β*	MF770571.1	CGGCAGATGTGACCTGCACA	CAGAGTAAAAGCCAGCAGAAG
*p65nf* *κb*	KY751029.1	ACTACGTGGGTCATGCTCGG	TGCTGCAGGTTCCGTTCTCA
*myd88*	MH778540.1	GAGGTGTAAGAGGATGGTGGTT	TGTGGAGGGTCTGGTGTAGTCA
Apoptosis-related genes			
*caspase3*	KY072821	TCTACGGCACAGATGGATCC	TGTGTGCCTTCTGACTCACT
*caspase9*	KY053837	TTCTGTCGAGGGGCATCTTT	AGGAACGGGTACAGGAACAG
*apaf1*	KY053839	ACCGCCAAATAGCAACCTG	CTGCTCCTCGTGCTCAACAT
*p53*	HQ419002.1	TGGGAAAACGAAGAGCAAAT	ATCGGAGGTGACAGGGACA
*baxa*	KY072819	TCGGAGACGAACTGGACAAC	TCGACAAGCAAAGTAGAAAAGC
*bcl2*	KY053838.1	TTTCACCGCCGTGATCG	CCAACTTGCTATGTTGTCCACC
JAK-STAT signaling			
*jak1*	XM_027146298.1	CGGAACCTCTGAAAACAAGTC	TGTCCCCGAGAAAAGAGATAG
*stat3*	KP342389.1	ACTCCGGTTGCCAAATCACT	CCTCATTCCACAGAGCCAGTAT
*stat5*	KP342392.1	ATCACCAGACCACAGGCACC	CACCACGACAGGCAAAGACAG
Autophagy			
*becn1*	KY062770	CTCAACTGGACCGCCTGAAGAAA	CACTCCACAGGAACGCTGGGTAAT
*ulk1a*	KY404999	GCGATTAAACAGGGCAAACTCTATCC	GCTGTGATGTTGTTCATTCGGTCC
*atg5*	KY062771	CAGAACCGTTTTATCTTCTCCTACCG	CGTCTACATCTTCAGCTTTCACGACTT
*lc3β*	KY062774	CCTGACCACGTCAACATGAGCGAACT	GGAAATGGCGGCAGACACGGAGA
Antioxidant enzymes			
*cat*	NW0208479561 1	TCTGTTCCCGTCCTTCATCC	ATATCCGTCAGGCAATCCAC
*gpx*	XR_003438442.1	ATCTACATTGGCTTGGAAAC	GAAAGTAGGGACTGAGGTGA
*Cu/Zn-sododDsod*	KT751173.1	GGCGGAGATGATGAAAGT	GAAAGGAAGCGGTGAAAC
*Mn-sod*	KT751172.1	TGGTGCTTGCTATGGTGA	GGCTTGAATCCCTTGCTG

*ef1α*	KR061492.1	GTCTGGAGATGCTGCCATTG	AGCCTTCTTCTCAACGCTCT

*il-8*, interleukin8; *il-1β*, interleukin 1 beta; *p65nfκb*, p65-nuclear factor kappa b; *myd88*, myeloid differentiation primary response 88; *caspase3*, cysteine-aspartic acid protease 3; *caspase9*, cysteine-aspartic acid protease 9; *apaf1*, apoptotic protease-activating factor 1; *p53*, tumor protein 53; *baxa*, *bcl2* associated X protein; *bcl2*, b-cell cll/lymphoma 2; *jak1*, janus kinase 1; *stat3*, signal transducer and activator of transcription 3; *stat5*, signal transducer and activator of transcription 5; *becn1*, *beclin1*; *ulk1a*, unc-51 like autophagy activating kinase 1a; *atg5*, autophagy related 5; *lc3β*, light chain 3 beta; *cat*, catalase; *gpx*, glutathione peroxidas; *Cu/Zn-sod*, Cu/Zn-superoxide dismutase; *Mn-sod*, Mn-superoxide dismutase; *ef-1α*, elongation factor 1 alpha.

**Table 3:** Effects of glutamine on weight gain rate, specific growth rate, feed conversion rate of yellow catfish, and its survival rate after challenge with *Flavobacterium columnare*. All data were analyzed using student’s *t*-test [56,59].

	IBW (g)	FBW (g)	WGR (%)	SGR (%/day)	FCR	SR (%)
Con	4.61 ± 0.02	28.04 ± 0.30	507.74 ± 6.84	1.40 ± 0.01	1.07 ± 0.01	73 ± 4
Gln	4.66 ± 0.02	29.38 ± 0.09	530.44 ± 2.29	1.43 ± 0.00	0.94 ± 0.04	92 ± 4
*p*	0.159	0.012	0.035	0.036	0.042	0.033

## Data Availability

Data are available from the corresponding author if requested.

## Abbreviations

DAMP	damage-associated molecular patterns
FA	fatty acids
FBW	final body weight
FCR	feed conversion ratio
FSGD	fish specific genome duplication
Gln	glutamine
MAPK	mitogen-activated protein kinase
mTOR	mechanistic target of rapamycin
ROS	reactive oxygen species
SGR	specific growth rate
TAOC	total antioxidant capacity
TG	triglyceride
WGR	weight gain ratio
